# A Case Report of Spontaneous Pneumomediastinum From an Unusual Cause: Baritone Practice

**DOI:** 10.7759/cureus.47289

**Published:** 2023-10-18

**Authors:** Patrick Meloy, Amit Bhambri, Iyesatta M Emeli

**Affiliations:** 1 Emergency Medicine, Emory University School of Medicine, Atlanta, USA; 2 Emergency Medicine, Swedish Hospital - Part of NorthShore University HealthSystem, Chicago, USA

**Keywords:** chest pain, spontaneous pneumomediastinum, cardiothoracic surgery, pulmonary barotrauma, emergency medicine, case report

## Abstract

Spontaneous pneumomediastinum (SPM) is a rare but potentially life-threatening clinical entity in which free air is introduced into the mediastinum. It most commonly presents in young males and has an incidence of approximately 0.002% of the general population. Symptoms include sudden onset chest pain, dyspnea, neck pain, vomiting, and odynophagia. Physical examination usually reveals subcutaneous emphysema, hoarse voice, tachycardia, tachypnea, and occasionally a Hamman’s sign, which is a mediastinal “crunch” sound heard on cardiac auscultation. We present a case of an 18-year-old male baritone player who presented to the ED with chest pain and odynophagia shortly after waking up one morning. The patient’s chest radiograph (CXR) revealed free air in the mediastinum with subcutaneous air tracking into the soft tissues of the neck and supraclavicular region. CT of the chest with contrast esophagram confirmed the diagnosis of primary SPM. The cause of his condition was likely due to barotrauma secondary to playing the baritone in his marching band. He had no evidence of esophageal injury or infectious process which further supports the diagnosis of primary SPM. After an extensive workup, the patient was discharged from the ED with instructions on rest, analgesia, and antitussives as needed. Evaluation of chest pain patients in the ED should include a CXR, in addition to other indicated tests, to rule out this potentially debilitating condition. Fortunately, though SPM is potentially life-threatening, most cases resolve spontaneously without surgical intervention.

## Introduction

Spontaneous pneumomediastinum (SPM) is a rare clinical entity thought to occur in approximately 0.002% of the population, resulting in approximately 1 in 30,000 ED visits in the United States [1,2]. SPM tends to occur during activities that increase intrathoracic pressure, such as forceful coughing or vomiting, intense physical activity, childbirth, or performing the Valsalva maneuver [1-3]. This condition is usually seen in young males who experience an acute onset of chest pain, dyspnea, odynophagia, and/or voice changes [2,4]. Physical examination frequently reveals subcutaneous emphysema, hoarse voice, tachycardia, tachypnea, and occasionally a “Hamman’s sign,” which is a crunching sound heard on cardiac auscultation [1-5]. While patients with SPM typically fare well, the initial investigation should be extensive in order to rule out serious conditions such as esophageal rupture, i.e., Boerhaave syndrome, ruptured viscus, or blunt trauma [2]. Typical imaging modalities include plain film radiographs of the chest, CT scans, esophagrams, and endoscopy [2]. Treatment is usually symptomatic with rest, analgesics, and the use of oxygen if needed [2]. Here, we present a case of an 18-year-old male patient who suffered an SPM as a result of baritone practice in his marching band. This appears to be the first report of its kind. His condition was initially picked up on a routine chest radiograph (CXR) and subsequently confirmed with CT of the chest with and without IV contrast. An esophagram was also obtained in order to evaluate for esophageal injury. The patient remained stable through his ED course and was eventually discharged home after consultation with cardiothoracic surgery.

## Case presentation

An 18-year-old male patient with no prior medical history presented to the ED with complaints of chest pain and painful swallowing. His symptoms started 12 hours prior to arrival when he woke up from sleep that morning. The patient denied any upper respiratory infection symptoms such as rhinorrhea, sinus pain/pressure, or fevers/chills. He denied using tobacco, alcohol, or any illicit substances. He is a college student who lives in a dormitory and plays the baritone on his school's marching band.

On examination, the patient appeared comfortable and in no acute distress. Vital signs on arrival were blood pressure 126/73 mm Hg, heart rate 97 beats per minute, respiratory rate 16 breaths per minute, temperature 36.9℃ (98.4℉), and oxygen saturation 100% on room air. The patient’s oropharyngeal exam revealed mild tonsillar erythema bilaterally, without edema or exudate. There was no anterior or posterior cervical lymphadenopathy. The trachea appeared midline and there was no stridor. The patient’s lung sounds were clear bilaterally, and heart tones were unremarkable, without any murmurs, gallops, or rubs. There was no palpable crepitus, nor signs of trauma such as ecchymosis or tenderness of the chest wall. The rest of the patient’s physical exam did not reveal any acute findings. A 12-lead EKG was obtained which revealed an RSR pattern in V1 and V2 and T-wave inversions in V1 and V2, which are consistent with normal pediatric EKG findings, expected given his age.

**Figure 1:**
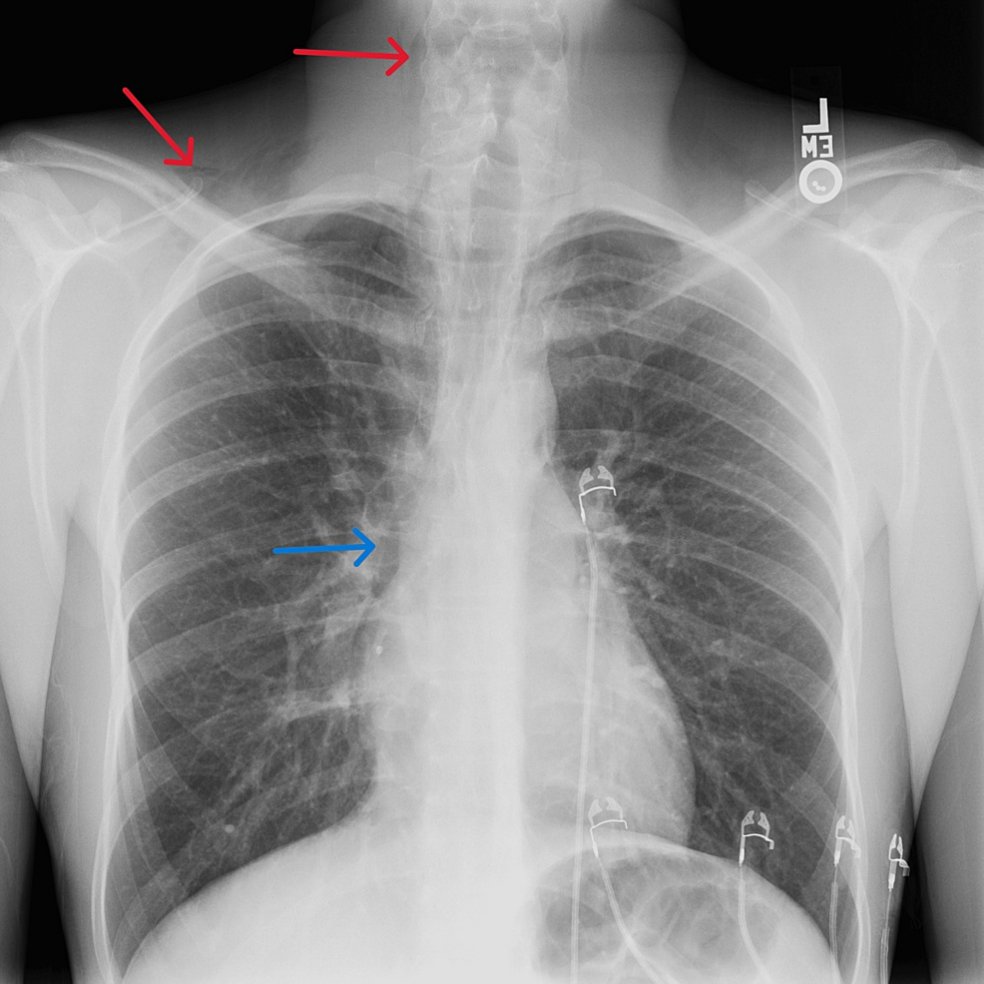
Anterior-posterior chest radiograph depicting pneumomediastinum (blue arrow) with associated subcutaneous emphysema in the lateral neck and supraclavicular spaces (red arrows)

The patient was treated symptomatically for a possible viral syndrome with IV fluids, 15 mg IV ketorolac, and 12 mg IV dexamethasone. Initial laboratory workup in the ED was unremarkable including a negative rapid strep swab, negative COVID-19, influenza and respiratory syncytial virus panel, normal electrolyte panel, and normal complete blood count. Subsequently, a two-view CXR was obtained which revealed the patient’s pneumomediastinum and associated subcutaneous emphysema of the neck and right supraclavicular area (Figure 1).

**Figure 2:**
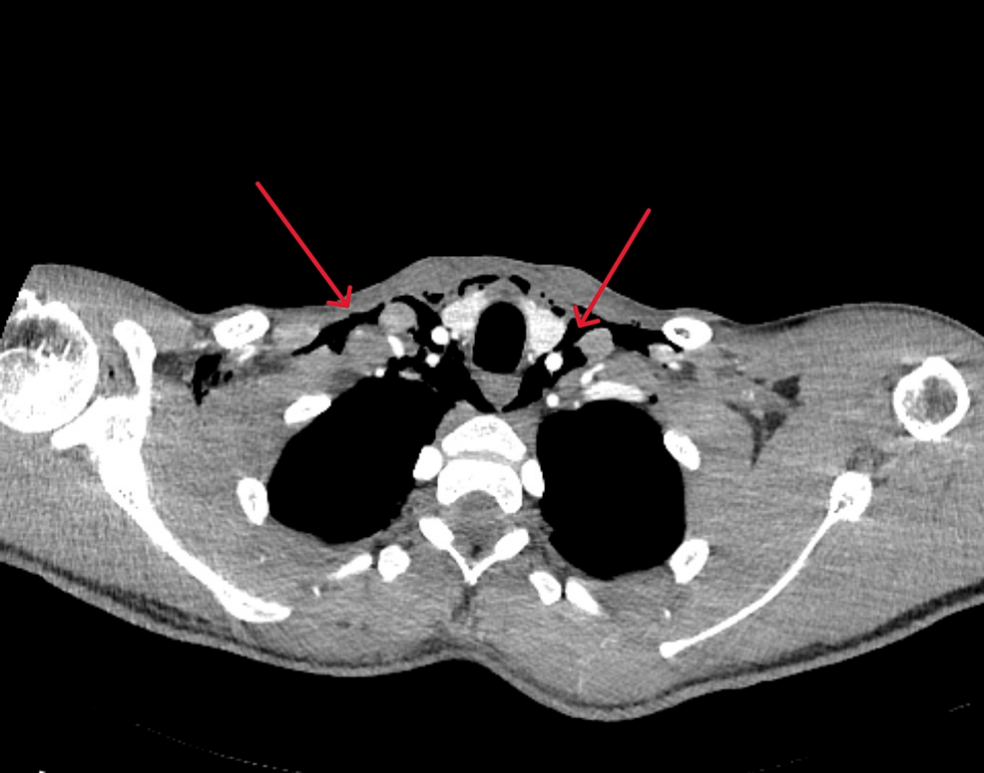
CT scan of the chest with IV contrast, in axial view, that indicates anterior and superior pneumomediastinum (red arrows)

**Figure 3:**
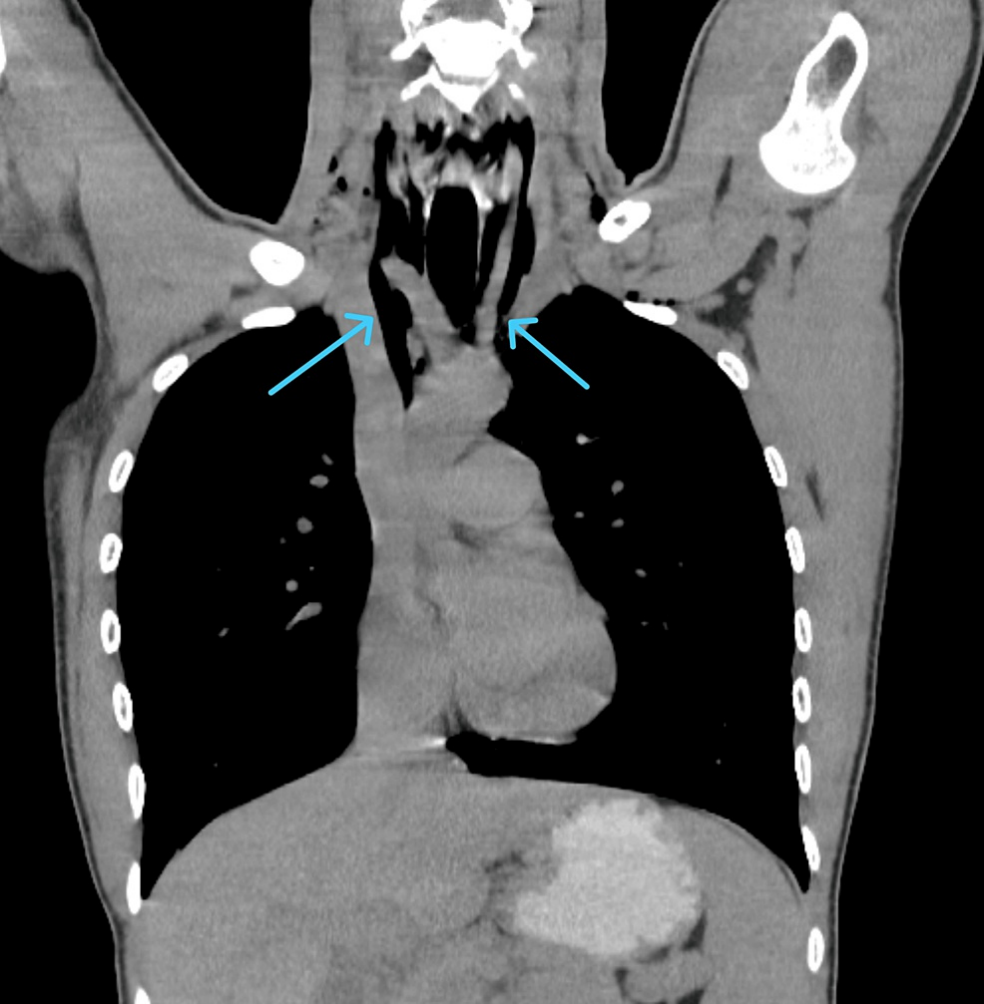
CT scan without IV contrast, in coronal view, which indicates superior pneumomediastinum (blue arrows)

Cardiothoracic surgery was immediately consulted and the patient underwent additional imaging, which included a CT scan of the chest with and without IV contrast, as well as a CT esophagram to rule out esophageal rupture (Figures 2-3).

After reviewing the case and imaging with cardiothoracic surgery, the patient was deemed stable for discharge with close outpatient follow-up, as he was not found to have any esophageal injuries or signs of infection. He was given instructions on symptomatic care at home. It was determined that his primary SPM was the result of barotrauma secondary to playing the baritone, which he was instructed to discontinue. He was referred to his primary care team for follow-up and was discharged home with prescriptions for analgesia.

## Discussion

Louis Virgil Hamman’s case series, published in 1939, was the first of its kind to characterize SPM [6]. The crunching sound heard during the cardiac cycle of affected patients accordingly bears his name - Hamman’s crunch or Hamman’s sign - and SPM is also known as Hamman’s syndrome [7]. Also in 1939, Charles Clifford Macklin, a pulmonologist, described the pathophysiology of the condition. SPM, he explained, results when a sudden increase in intrathoracic pressure leads to alveolar rupture and consequent air dissection and tracking along the bronchovascular sheaths and into the mediastinum [8]. This mechanism became known as the “Macklin effect” [7]. The patient in our case plays the baritone and this may have led to the Macklin effect and to his SPM. Since there is scant literature on SPM associated with the use of wind instruments [9], we believe our case study to be an unusual presentation of this condition.

Authors have also referred to SPM as primary SPM (PSPM), thereby distinguishing it from secondary pneumomediastinum [10]. SPM (or PSPM) is a self-limited condition with no traumatic or iatrogenic cause. In contrast, secondary pneumomediastinum is secondary to an obvious factor such as recent surgery, procedure, or trauma [11]. The distinction is important because secondary pneumomediastinum, unlike SPM, follows a morbid course with high mortality rates and prolonged recovery times [12].

Even though SPM typically follows a benign course, it remains important to exclude critical conditions, such as esophageal perforation, pneumothorax, and mediastinitis, hence, the extensive workup in the case of this patient. This type of resource-intense evaluation, often including invasive tests, is also highlighted in a 2021 comprehensive review of SPM published by Morgan et al. [10]. A total of 535 patients in the PubMed database were reviewed. Nearly all of the 535 patients, as one would expect, underwent chest X-rays, while 65% had CT scans, 35.6% esophagrams, 13% esophagogastroduodenoscopies, and 14.6% bronchoscopies. The hospital admission rate was 86.5%, with an average length of stay of 4.4 days, whereas in this case, the patient was discharged directly from the ED. There were no fatalities [10].

Since approximately 10% of SPM patients have a concurrent pneumothorax [10], diagnostic workup to exclude this potential life-threatening process should always be undertaken. Further tests are often undertaken because of the difficulty in discriminating between esophageal rupture and SPM. Finally, the SPM recurrence rate has been estimated at 0-4.6% [13], and patients should always be made aware of this future risk.

## Conclusions

In conclusion, the case described here highlights an important risk factor for SPM that has only been minimally reported in the literature. Emergency physicians and general practitioners should be aware of the potential risk of SPM with the use of wind instruments in their evaluation of musicians with chest pain or dyspnea. Patients found to have SPM should undergo additional testing, which may include a CT scan of the chest, esophagram, bronchoscopy, and/or esophagogastroduodenoscopy, to exclude esophageal rupture, pneumothorax, mediastinitis, and other life-threatening conditions. Patients stable for outpatient follow-up should be informed of the risk of recurrence and to modify risk factors accordingly.
